# Carcass Composition, Meat Quality, and Digestive and Skeletal Traits of Muscovy and Pekin Broiler Ducks

**DOI:** 10.3390/ani16121918

**Published:** 2026-06-20

**Authors:** Marcin Wegner, Dariusz Kokoszyński, Kamil Stęczny, Mohamed Saleh, Marek Kotowicz, Joanna Żochowska-Kujawska, Dariusz Piwczyński

**Affiliations:** 1 Boehringer-Ingelheim, 00-728 Warsaw, Poland; 2 Department of Animal Breeding and Nutrition, Bydgoszcz University of Science and Technology, 85-084 Bydgoszcz, Poland; kokoszynski@gmail.com (D.K.); kamil.steczny@o2.pl (K.S.); 3 Department of Poultry and Animal Production, Faculty of Agriculture, Sohag University, Sohag 82524, Egypt; mohamed.saleh@agr.sohag.edu.eg; 4 Department of Meat Science, West Pomeranian University of Technology in Szczecin, 71-550 Szczecin, Poland; marek.kotowicz@zut.edu.pl (M.K.); joanna.zochowska-kujawska@zut.edu.pl (J.Ż.-K.); 5 Department of Animal Biotechnology and Genetics, Bydgoszcz University of Science and Technology, 85-084 Bydgoszcz, Poland; darekp@pbs.edu.pl

**Keywords:** Pekin duck, Muscovy duck, carcass composition, meat quality, digestive tract morphometry, leg bone dimensions

## Abstract

The aim of this study was to compare carcass composition, meat quality, digestive tract morphometry, and leg bone dimensions of Pekin and Muscovy ducks. The study involved 40 birds, including 10 males and 10 females from each genotype, reared to market age. Carcass traits, physicochemical properties of breast and leg muscles, texture parameters, internal organ development, intestinal measurements, and selected dimensions of the femur and tibia were evaluated. The results demonstrated a significant effect of duck genotype (*p* < 0.05) on carcass weight, dressing percentage, and the proportion of neck, wings, and skin with subcutaneous fat. Genotype also affected meat color (L*, a*, b*), intramuscular fat and collagen content, cooking loss, pH, electrical conductivity, and selected texture parameters of breast muscles. Differences were also observed in the mass and proportion of internal organs, most intestinal morphometric traits, and selected bone measurements. Sex had a significant effect on body and carcass weight, selected meat quality traits, intestinal measurements, and leg bone dimensions, with males generally showing greater body size and more developed skeletal structures. Significant interactions between genotype and sex were observed for several analyzed traits. The findings indicate that both genotype and sex substantially affect slaughter traits and meat quality characteristics of ducks.

## Simple Summary

This study compared carcass composition, meat quality, digestive tract morphometry, and leg bone dimensions of Pekin and Muscovy ducks. Forty ducks (10 males and 10 females from each breed) were evaluated at market age. The results showed that both breed and sex significantly affected carcass traits, meat quality, internal organ development, intestinal measurements, and bone dimensions. Muscovy ducks were characterized by a higher proportion of breast and leg muscles, higher protein content, lower intramuscular fat content, and greater springiness of the breast muscles. In contrast, Pekin ducks showed higher fat deposition, lighter meat color, higher intramuscular fat content, and greater cooking loss. Males generally had higher body and carcass weights and larger leg bone dimensions than females. These findings have practical implications for commercial duck production systems, indicating that Muscovy ducks may be more suitable for producing leaner meat with a higher protein content, while Pekin ducks may be preferred where higher intramuscular fat content and different sensory qualities are desired. The results also demonstrate that both genotype and sex should be considered in breeding and management strategies aimed at optimizing carcass yield and meat quality.

## 1. Introduction

Ducks are among the oldest poultry species domesticated by humans and constitute an important component of global poultry meat production. According to FAO data [1], global duck meat production reached 7.3 million tonnes in 2024, accounting for 5.0% of total poultry meat production worldwide. Of particular economic importance are Pekin ducks and Muscovy ducks (*Cairina moschata*), which differ significantly in terms of growth rate, carcass characteristics, and meat quality traits [2]. The Pekin duck originated from the mallard (*Anas platyrhynchos domesticus*) and is currently the most widely reared meat-type duck in the world [3]. In contrast, the Muscovy duck originated in Central and South America, where it was domesticated by indigenous populations many centuries ago [2]. Pekin ducks are characterized by rapid growth and can reach a body weight of 2.5 kg by 5 weeks of age [4]. Compared with Pekin ducks, Muscovy ducks exhibit a slower growth rate and lower feed efficiency, which results in higher production costs for this breed. The increased production costs translate into a higher market price of Muscovy duck carcasses compared with those of Pekin ducks. This makes Muscovy duck carcasses less price-competitive in the consumer market than those of Pekin ducks. Moreover, Muscovy ducks have greater nutritional and environmental requirements, which further reduce the economic efficiency of their production [5]. In intensive production systems, females are usually slaughtered earlier than males due to pronounced sexual dimorphism and differences in growth rate [6]. Moreover, Muscovy ducks produce fewer eggs annually, require a longer incubation period, and generally exhibit poorer hatchability than Pekin ducks [7,8]. A characteristic feature of Muscovy ducks is their pronounced sexual dimorphism [7]. Their advantages include a higher proportion of breast and leg muscles, a lower proportion of skin with subcutaneous fat and abdominal fat, as well as superior meat quality compared with that of Pekin ducks [5,7,9,10,11].

Currently, duck meat is gaining increasing consumer interest due to its nutritional value, distinctive flavor, and favorable technological properties [11]. Duck meat production continues to grow, particularly in Asia and Europe, with France, Poland, and Hungary being the main European producers [12]. China remains the largest producer of duck meat, accounting for approximately 71.6% of global production, with more than 4.4 billion ducks produced in 2025 [13,14]. Duck meat is valued for its high content of protein, minerals, and B vitamins and is characterized by a favorable fatty acid profile, with a high proportion of unsaturated fatty acids [15,16,17,18]. Compared with other poultry species, it generally contains higher levels of intramuscular fat, contributing to improved juiciness and sensory quality [19].

Meat quality is strongly influenced by physicochemical traits such as pH, water-holding capacity, cooking loss, texture, and color, which are key determinants of consumer acceptance [15,20,21,22]. Excessive water loss during thermal processing negatively affects technological yield, meat juiciness, and nutrient retention [23,24,25]. The quality of poultry meat depends on numerous factors, including genotype, slaughter age, sex, feeding strategy, and rearing conditions [19,26]. Muscovy and Pekin ducks differ in growth performance, the proportion of breast and leg muscles in the carcass, fat deposition, and physicochemical properties of meat [27,28]. Muscovy ducks are generally characterized by a higher proportion of muscles and lower carcass fatness, whereas Pekin ducks show a higher fat content and juicier meat [27]. These differences may affect both the dietary value and technological suitability of the meat.

The digestive system is a key component of the body’s defense system and the primary site of digestion and nutrient absorption from the diet [29,30]. The most intensive growth of the small intestine occurs during early life, whereas its development rate gradually decreases with age [31]. Rapid intestinal development in the first weeks of life is associated with the need to ensure efficient digestion and absorption of nutrients required for intensive growth. According to Watkins et al. [32], the digestive system of Pekin ducks reaches morphological and functional maturity after 7 weeks of age. After this period, the growth rate of digestive organs decreases, and dietary energy is increasingly directed toward muscle and fat deposition. However, the development and functioning of the digestive tract may be influenced by several factors, including genotype, age, diet composition, environmental conditions, and the production system [19,30,31,32]. Differences between duck genotypes may affect both the size of individual intestinal segments and nutrient utilization efficiency, directly influencing production performance and carcass quality.

Available literature provides relatively limited information comparing carcass composition and meat quality of Muscovy and Pekin ducks reared to market age. Most previous studies have focused mainly on production performance [10,33] or selected physicochemical properties of meat [33,34]. Moreover, the available reports usually evaluate individual groups of traits separately and rarely consider the simultaneous effects of genotype and sex. To our knowledge, no previous study has comprehensively compared carcass composition, meat quality characteristics, digestive tract morphometry, and leg bone dimensions in Muscovy and Pekin ducks within a single experimental framework. Therefore, the present study provides a more integrated assessment of phenotypic differences between these two economically important duck genotypes and extends current knowledge on the relationships between carcass traits, meat quality, gastrointestinal development, and skeletal characteristics. Accordingly, the aim of the present study was to compare carcass composition, meat quality, digestive tract morphometry, and leg bone dimensions in Muscovy and Pekin ducks at market age. The study evaluated carcass characteristics, the proportion of individual anatomical components, and selected physicochemical properties of meat important for its technological and consumer value. The effect of sex on carcass parameters and meat quality traits in both duck genotypes was also assessed.

## 2. Materials and Methods

### 2.1. Materials

The experimental material consisted of 80 ducks (40 Muscovy and 40 Pekin), which were divided randomly into four pens with an area of 12 m^2^ according to genotype and sex. The ducks were reared on straw litter in a windowless building without access to an outdoor run. During the rearing period, birds were maintained under a 16 h light: 8 h dark photoperiod, except for the first three days of life when continuous lighting was provided. During the first 3 weeks of life, the temperature in the building was maintained at 23–24 °C, while under the heat lamps it ranged from 30 to 32 °C. In the following weeks, the temperature was gradually reduced by 2–3 °C per week under the heaters and by 1 °C per week in the rearing area. From the fourth week of life, the temperature was maintained at 21 ± 1 °C. Throughout the entire rearing period, relative humidity in the building ranged from 60 to 70%. During the experiment, birds had ad libitum access to feed and water. From 1 to 21 days of age, ducks were fed a complete starter diet containing 20.62% crude protein and 12.19 MJ of metabolizable energy per kg of feed. From 22 days of age until the end of the rearing period, i.e., day 49 for Pekin ducks, day 70 for female Muscovy ducks, and day 84 for male Muscovy ducks, the birds received a complete grower/finisher pelleted diet. This diet contained 17.59% crude protein and 12.68 MJ of metabolizable energy per kg of feed.

### 2.2. Carcass Analysis

At the end of the rearing period (day 49 for Pekin ducks, day 70 for female Muscovy ducks, and day 84 for male Muscovy ducks), 10 females and 10 males with body weights closest to the arithmetic mean were selected from each genotype and sex group. Prior to slaughter, birds were subjected to a 10 h feed withdrawal period. The ducks were then slaughtered manually following stunning by a blow to the head with a baton and exsanguination through severing of the neck blood vessels. After slaughter, the birds were defeathered and eviscerated, and the carcasses, together with giblets (heart, gizzard, and liver) and viscera, were chilled for 18 h in a refrigerated cabinet (Hendi, Gądki, Poland) at 4 °C. After chilling, the carcasses were weighed and dissected according to the method described by Ziołecki and Doruchowski [35]. During dissection, breast muscles (musculus pectoralis major), leg muscles (thigh and drumstick muscles), skin with subcutaneous fat, abdominal fat, wings, neck, and carcass remains were separated from each carcass and weighed using a PS 1000.R2 scale (Radwag, Radom, Poland) with an accuracy of 0.1 g. Subsequently, the percentage share of each component in the carcass was calculated according to the following formula: weight of the carcass component/cold eviscerated carcass weight × 100%. Slaughter yield was also calculated as the ratio of eviscerated carcass weight to pre-slaughter body weight × 100%.

### 2.3. Physicochemical Analyses

Before dissection and 24 h postmortem, pH and electrical conductivity of the superficial breast and thigh muscles were determined (pH_24_ and EC_24_). Muscle acidity was measured using a CX-701 pH meter equipped with a glass electrode mounted in a steel knife (Elmetron, Zabrze, Poland). Prior to analysis, the device was calibrated using buffer solutions of pH 7.0 and 4.0. The results were recorded with an accuracy of 0.01 pH units. Electrical conductivity of the meat (EC_24_) was determined using an LF-Star CPU conductivity meter (Ingenieurbüro R. Matthäus, Nobitz, Germany) with an accuracy of 0.1 mS/cm. The measurements of pH and electrical conductivity were performed in duplicate for each muscle. The electrode was inserted into the breast and thigh muscles at a 90° angle to the direction of the muscle fibers. The color of breast and leg muscles was evaluated using a MINOLTA CR 400 colorimeter (Konica Minolta, Chiyodaku, Japan). Measurements were performed in the CIE L*a*b* color system, determining the following parameters: L* (lightness), a* (redness), and b* (yellowness). The measured area was 50 mm in diameter. The was calibrated against a CR310 white reference tile (Y = 92.80, x = 0.3175, y = 0.3333). The basic chemical composition of the meat, including water, protein, fat, and collagen content, was determined using near-infrared transmission spectroscopy (NIRS) with a FoodScan analyzer (FoodScan, Hillerød, Denmark). The application of a single FOSS Artificial Neural Network (ANN) calibration, capable of analyzing all types of meat products, enabled accurate determination of the studied parameters of the basic chemical composition [36]. From each carcass, 90 g samples of breast muscles and 90 g samples of leg muscles were collected and homogenized using an electric meat grinder (Zelmer, Rzeszów, Poland). To determine cooking loss, samples of breast and leg muscles weighing 20 ± 2 g were prepared. Each sample was wrapped in gauze and subjected to heat treatment in a water bath at 85 °C for 10 min. After heating, the samples were cooled for 30 min in a refrigerated cabinet at 2 °C. The samples were then reweighed using an electronic scale. Cooking loss was calculated based on the difference between the sample weight before and after heat treatment and expressed as a percentage of the initial sample weight [37].

### 2.4. Texture Analysis

Prior to analysis, the samples were stored in a cold store at 3 °C for approximately 12 h. Immediately prior to analysis, the samples were removed from the cold store and left at room temperature for approximately 2 h. During this time, they were protected from moisture loss and surface drying with food-grade plastic film. To evaluate textural properties, 40 samples of the pectoralis major muscle were analyzed, including 20 samples from each genotype, with 10 males and 10 females per group. Meat texture was assessed using texture profile analysis (TPA) and the Warner–Bratzler shear force (WB) test with a TA.XT Plus texture analyzer (Stable Micro Systems, Godalming, UK) [38]. In the TPA, a cylindrical probe with a diameter of 0.61 cm was used to compress the samples twice parallel to the direction of the muscle fibers to 80% of the initial sample height (16 mm). The samples were compressed parallel to the direction of the muscle fibers. Measurements were performed at a crosshead speed of 50 mm·min^−1^ using a 50 N load cell. Based on the obtained force–deformation curve, the following texture parameters were determined: hardness, cohesiveness, springiness, chewiness, and gumminess [38]. For the Warner–Bratzler test, muscle samples measuring approximately 1.0 × 1.0 × 2.0 cm were cut perpendicular to the muscle fibers using a triangular blade. Measurements were conducted at a crosshead speed of 50 mm·min^−1^ with a 500-N load cell [38]. Both the TPA and WB tests were repeated 15 times for each muscle sample.

### 2.5. Anatomical Analysis

The weights of the proventriculus, gizzard, liver, heart, and spleen were determined using an electronic balance WPS 210/C (Radwag, Radom, Poland) with an accuracy of 0.01 g. The percentage share of each organ in body weight was calculated according to the following formula: organ weight/body weight × 100%. Subsequently, the lengths of individual sections of the small intestine, including the duodenum, jejunum, and ileum, as well as the lengths of the caeca and colon, were measured in their natural (relaxed) state using a measuring tape with an accuracy of 1 mm. Measurements were taken at three points along each intestinal segment: at the beginning, in the middle of the segment, and at the end of the examined section.

### 2.6. Measurements of Leg Bone Dimensions

Measurements of the hind limb bones were performed after carcass dissection using an electronic caliper with an accuracy of 0.01 mm. For the femur, the following parameters were determined: GL—greatest length, ML—medial length, GB—greatest breadth of the proximal end, GD—greatest depth of the proximal end, SM—smallest breadth of the shaft, GC—greatest breadth of the distal end, and GE—greatest depth of the distal end. For the tibia, the following measurements were recorded: GL—greatest length, AL—axial length, GD—greatest diagonal of the proximal end, SB—smallest breadth of the shaft, SD—greatest breadth of the distal end, and DD—greatest depth of the distal end. Measurements of the femur and tibia were performed according to the method described by von den Driesch [39].

### 2.7. Statistical Analysis

Data obtained during the study concerning carcass weight and composition, basic chemical composition, physicochemical and textural traits of meat, gastrointestinal tract morphometry, and femur and tibia bone dimensions were subjected to statistical analysis. For each analyzed trait, arithmetic means were calculated for the experimental factors (breed or sex), and SEM values (standard error of the mean) were determined for both groups combined. The influence of breed and sex on the examined traits of ducks was investigated using a two-way analysis of variance. Finally, the following linear model was used: *Y_ijk_* = *m* + *a_i_* + *b_j_* + (*a* × *b*)*_ij_* + *e_ijk_,* where *Y*_ijk_ is the value of the analyzed trait, m is the overall mean for the tested trait, ai is the effect of *_i_*-th genotype, b*_j_* is the effect of *_j_*-th sex, (*a* × *b*)_ij_ is the genotype by sex interaction, *e_ijk_* is the random error. Statistical calculations were performed using SAS software version 9.4 [40]. The significance of differences between breeds and between males and females at *p* < 0.05 was verified using Tukey’s post hoc test.

## 3. Results

### 3.1. Carcass Composition

Analysis of the effects of genotype and sex on body weight, carcass weight, dressing percentage, and the percentage share of individual carcass components (Table 1) revealed a significant (*p* < 0.05) effect of genotype on the percentage share of the neck, skin with subcutaneous fat, and wings. The percentage share of wings was higher in both female and male Muscovy ducks, while the proportion of the neck was higher in female and male Pekin ducks (*p* < 0.001). A higher percentage of skin with subcutaneous fat was observed in male Pekin ducks compared with male Muscovy ducks (*p* = 0.034). No effect of duck genotype was found for the remaining analyzed traits, including body weight, carcass weight, dressing percentage, the percentage share of breast muscles, leg muscles, abdominal fat, and carcass remainder (*p* = 0.063–0.415). The body weight of male Muscovy ducks was significantly higher than that of females (*p* < 0.001). Higher carcass weight was also observed in male Muscovy and Pekin ducks compared with females (*p* < 0.001).

The percentage proportion of the neck without skin was higher in male Muscovy ducks (*p* = 0.031). Sex had no effect on the remaining analyzed traits, including dressing percentage, percentage of breast muscles, leg muscles, abdominal fat, skin with subcutaneous fat and carcass remainder (*p* = 0.155–0.826). An interaction between genotype and sex was observed for body weight, carcass weight, percentage of leg muscles, and skin with subcutaneous fat (*p* < 0.001–0.014).

### 3.2. Basic Chemical Composition

Analysis of the basic chemical composition of breast and leg muscles (Table 2) revealed an effect of duck genotype on all analyzed traits, except for protein and water content in the breast muscle (*p* = 0.101 and 0.324, respectively). Intramuscular fat content in both breast and leg muscles was higher in female and male Pekin ducks, whereas a higher water content in leg muscles was observed in female and male Muscovy ducks (*p* < 0.001). Protein content in leg muscles was higher in male Muscovy ducks, while collagen content was lower than that in male Pekin ducks (*p* = 0.008 and 0.011, respectively). Collagen content in breast muscles was higher in female Pekin ducks than in female Muscovy ducks within the same sex group (*p* = 0.002). Sex had a significant effect on intramuscular fat and water content in leg muscles. Higher fat content in leg muscles was found in female Muscovy ducks, whereas a higher water content was observed in female Pekin ducks compared to males within the same breed (*p* < 0.001). No effect of sex was observed for the remaining analyzed traits (*p* = 0.066–0.726). An interaction between duck genotype and sex was also observed for fat content in breast and leg muscles, protein content in breast muscles, and water and collagen content in leg muscles (*p* < 0.001–0.003).

### 3.3. Physicochemical Properties

Analysis of pH and electrical conductivity of breast muscles showed no significant effect (*p* < 0.05) of duck genotype on the analyzed traits (Table 3). However, a significant effect of breed was observed for pH, EC, and cooking loss of leg muscles, as well as for cooking loss of breast muscles (*p* < 0.001–0.033). Higher pH values in leg muscles were recorded in female Pekin ducks. Likewise, cooking loss in both breast and leg muscles was higher in both female and male Pekin ducks compared with Muscovy ducks. In contrast, Muscovy ducks (both males and females) showed higher electrical conductivity in leg muscles. Sex had an effect on the cooking loss of breast muscles. Female Muscovy ducks had lower cooking loss in breast muscles compared to males (*p* = 0.035), whereas no such differences were observed between sexes in Pekin ducks. An interaction between genotype and sex was also observed for the pH of breast muscles and electrical conductivity of leg muscles (*p* = 0.009 and 0.020, respectively). Analysis of breast and leg muscle color revealed a significant effect of duck breed on all analyzed traits (L*, a*, and b*) (Table 4). Both male and female Pekin ducks showed higher L*, a*, and b* values in breast and leg muscles compared to Muscovy ducks (*p* < 0.001–0.012). Sex affected the L* value of breast muscles. Female Muscovy ducks exhibited lower L* values in breast muscles and lower b* values in leg muscles compared to males. An interaction between genotype and sex was observed for the L* value of breast muscles (*p* = 0.002).

### 3.4. Texture of Breast Muscles

Analysis of texture traits (Table 5) of the breast muscle showed a significant effect of genotype on Warner–Bratzler shear force, hardness, gumminess, and springiness (*p* < 0.001–0.013). Breast muscles of both female and male Pekin ducks were characterized by higher shear force, hardness, and gumminess, whereas higher springiness was observed in Muscovy ducks of both sexes. Sex, regardless of breed, had no effect on the analyzed texture traits (*p* = 0.169–0.876). No interaction between genotype and sex was observed for the analyzed texture traits of the breast muscle (*p* = 0.433–0.989).

### 3.5. Internal Organ Weights

Analysis of the mass and percentage share of internal organs (Table 6) showed an effect of duck genotype on most analyzed traits, except for the mass and percentage share of the proventriculus and the percentage share of the spleen in body weight. Both female and male Pekin ducks were characterized by higher weights of the gizzard and liver, as well as a lower proportion of the heart in body weight (*p* < 0.001). Male Pekin ducks also showed lower weights of the heart and spleen, and a higher proportion of the gizzard and liver in body weight (*p* < 0.001–0.005). Sex affected almost all traits, except for gizzard weight and the proportion of the heart and spleen in body weight (*p* = 0.671–0.861). Female Muscovy ducks were characterized by lower weights of the heart and spleen, and a higher proportion of the gizzard, proventriculus, and liver compared to males (*p* < 0.001–0.033). In contrast, female Pekin ducks showed a lower proventriculus weight compared to males (*p* = 0.020). Interactions between genotype and sex were also observed for proventriculus, heart, and spleen weights, as well as for the proportion of the gizzard and proventriculus (*p* < 0.001–0.030).

Table 7 presents the effects of duck genotype and sex on the length and diameter of individual intestinal segments. Genotype had a significant effect (*p* < 0.05) on all analyzed traits except caeca diameter (*p* = 0.085). Both male and female Pekin ducks had longer duodenum, jejunum, ileum, and total intestine (*p* < 0.001). Female Pekin ducks also had a longer caeca, while males had a shorter colon compared with male Muscovy ducks (*p* < 0.001). In both Muscovy and Pekin ducks, males had a longer jejunum, ileum, colon, and total intestine compared to females (*p* < 0.001). Male Muscovy ducks also showed longer duodenum and caeca compared to females (*p* < 0.001, 0.022; respectively). Both male and female Pekin ducks exhibited larger diameters of the duodenum, jejunum, ileum, and colon compared to Muscovy ducks (*p* < 0.001–0.001). Sex affected duodenal diameter, which was larger in female Muscovy ducks compared to males (*p* = 0.019). Interactions between genotype and sex were also observed for jejunum, ileum, caeca, colon, and total intestine length, as well as colon diameter (*p* = 0.001–0.047).

### 3.6. Bone Measurements

Analysis of individual traits of the tibia and femur (Table 8) revealed a significant (*p* < 0.05) effect of duck genotype on the dimensions of GL, ML, GB, GD, and GE of the tibia, as well as GL, AL, and DD of the femur. Both male and female Pekin ducks were characterized by higher values of GE and GD of the tibia compared to Muscovy ducks of both sexes (*p* < 0.001). In contrast, higher GL and ML values were observed in female Pekin ducks compared with female Muscovy ducks and in male Muscovy ducks compared with male Pekin ducks (*p* < 0.001). A greater GB value of the tibia was also recorded in male Muscovy ducks compared to male Pekin ducks (*p* < 0.001). The effect of sex on tibia parameters was observed only in Muscovy ducks, where males showed higher values of all analyzed traits (GL, ML, GB, GD, SM, GC, and GE) (*p* < 0.001). A greater DD measurement of the femur was found in both male and female Pekin ducks, whereas female Pekin and male Muscovy ducks were characterized by higher GL and AL values of the femur (*p* < 0.001). Sex had no effect on femur dimensions in Pekin ducks, while a sex effect was observed only in Muscovy ducks, where males showed higher values of GL, AL, GD, SD, and DD compared to females (*p* < 0.001). Interactions between genotype and sex were also observed for all analyzed tibia and femur traits except SM in the tibia and SB in the femur (*p* < 0.001).

## 4. Discussion

The results obtained in this study confirm the significant influence of both genotype and sex on the carcass traits of ducks. It was shown that male Muscovy ducks had a higher carcass weight and greater slaughter yield compared to male Pekin ducks. Similar relationships were observed by Omojola [34], who demonstrated a higher slaughter yield in male Muscovy ducks (71.18%) compared to male Pekin ducks (66.67%). These results may stem from the pronounced sexual dimorphism present in Muscovy ducks and the greater ability of males of this genotype to deposit muscle tissue. It should also be noted that the observed differences in body weight between male and female Muscovy ducks may have been partially influenced by the slight age discrepancy between the groups (14 days). During this growth stage, ducks still exhibit rapid body mass gain, which could have contributed to the magnitude of the observed differences. Nevertheless, given the well-documented strong sexual dimorphism in Muscovy ducks, age effects are likely secondary to genotype- and sex-related growth patterns. In the conducted studies, the body weight of male Muscovy ducks was significantly higher than that of females, which is consistent with the latest findings of Makram et al. [41] and Edrova et al. [42], who showed a higher slaughter weight and carcass weight in male Muscovy ducks compared to females. The authors emphasized that sex is one of the main factors determining growth rate and musculature in Muscovy ducks. In this study, no effect of genotype on the percentage of breast muscles or leg muscles in the carcass was observed. These results are consistent with the observations of other authors [42,43,44,45]. In our research, the percentage of skin with subcutaneous fat was higher in male Pekin ducks than in male Muscovy ducks. These findings are in agreement with observations made by Makram et al. [41], who reported greater fat deposition in Pekin duck carcasses compared to Muscovy ducks. The authors pointed out that Muscovy ducks are less prone to subcutaneous fat deposition and have a higher proportion of muscle tissue. The greater wing proportion observed in Muscovy ducks and the greater neck proportion in Pekin ducks may be related to the different anatomical structures and selection directions of both genotypes. Muscovy ducks have a more elongated silhouette and a better-developed locomotor apparatus, which could influence the greater wing proportion in the carcass. Similar morphological differences between Muscovy and Pekin ducks were described by Różewicz and Kaszperuk [7]. Within each sex, a higher percentage of leg muscles was found in male Muscovy ducks, while females of this genotype had a greater percentage of skin with subcutaneous fat. Studies conducted on the same breed confirmed greater fat deposition in females (2.21% vs. 0.72%) and showed higher slaughter yields in males (68.56%) compared to females (64.03%) [43]. Edrova et al. [42] reached similar conclusions. In contrast, the present studies showed no effect of sex on slaughter yield or the percentage of skin with subcutaneous fat and carcass remainder, regardless of the duck genotype. The obtained results may be associated with the action of sex hormones, which influence muscle development rates and fat deposition. Testosterone promotes protein synthesis and muscle fiber hypertrophy, which may contribute to the greater carcass weight and muscle development observed in males. In contrast, estrogens are involved in lipid metabolism and may favor fat deposition, particularly in females. These physiological differences could partly explain the sex-related variation observed in carcass composition. The significant interaction between genotype and sex in terms of body weight, carcass weight, and the proportion of leg muscles and skin with subcutaneous fat indicates that the body’s response to genetic factors may vary between males and females. The results confirm the necessity of considering both genotype and sex when assessing the utility value of ducks and planning poultry meat production.

Analysis of the basic chemical composition revealed a higher intramuscular fat content in both breast and leg muscles of Pekin ducks compared with Muscovy ducks. These results are consistent with the findings of Chartrin et al. [28] and Saez et al. [46], who demonstrated a greater ability of Pekin ducks to deposit intramuscular fat in the breast muscles compared with Muscovy ducks. The authors suggested that these differences may result from distinct lipid metabolism mechanisms between the genotypes, particularly the greater activity of processes related to lipid transport and deposition in the muscles of Pekin ducks. Moreover, Muscovy ducks are characterized by a higher proportion of muscle tissue and a lower degree of carcass fatness, which contributes to their lower intramuscular fat content. In the study by Chartrin et al. [28], it was shown that Muscovy duck muscles contain more water and less fat compared with Pekin duck muscles. These findings were also confirmed in the present study, where a higher water content in leg muscles was observed in both male and female Muscovy ducks. This relationship is probably associated with the inverse correlation between fat and water content in muscles, since increased fat deposition usually results in a lower proportion of water in muscle tissue. In the present study, a higher protein content in leg muscles was found in male Muscovy ducks. These results confirm earlier observations by Kokoszyński et al. [5], who indicated that Muscovy duck meat is characterized by higher nutritional value and greater protein concentration compared with Pekin duck meat. The higher protein content may be associated with a greater proportion of muscle tissue and lower fatness in Muscovy ducks. These differences may also reflect genotype-specific metabolic strategies. Muscovy ducks appear to allocate a greater proportion of nutrients toward lean tissue accretion, whereas Pekin ducks exhibit a higher propensity for lipid deposition. Such differences in nutrient partitioning may be linked to genetic variation in energy metabolism and muscle growth regulation. The observed higher collagen content in the breast muscles of female Pekin ducks and lower collagen content in the leg muscles of male Muscovy ducks may indicate differences in connective tissue structure between the analyzed genotypes. According to Le Bihan-Duval et al. [22], collagen content in poultry muscles depends on genotype, muscle activity, and bird growth rate. A higher collagen content may contribute to greater meat toughness and reduced tenderness. Duck sex mainly affected fat and water content in leg muscles. Female Muscovy ducks were characterized by higher intramuscular fat content, whereas female Pekin ducks had higher water content than males of the same genotype. Similar relationships were described by Houessionon et al. [43] and Edrova et al. [42], who demonstrated greater fat deposition in female Muscovy ducks compared with males. The lack of an effect of sex on most of the remaining analyzed parameters may indicate that the basic chemical composition of meat is more strongly determined by genotype than by sex.

Higher cooking loss values observed in Pekin ducks may indicate a lower water-holding capacity of the meat during heat treatment. Similar relationships were reported by Baéza et al. [27] and Chartrin et al. [28], who demonstrated that Pekin duck meat is characterized by greater fatness and higher cooking losses compared with Muscovy ducks. The higher pH value of leg muscles in female Pekin ducks may indicate a slower postmortem glycolysis process and lower muscle acidification after slaughter. In turn, the higher electrical conductivity of leg muscles in Muscovy ducks may be related to a greater degree of cell membrane damage and a more intensive release of electrolytes into the extracellular space. Similar relationships between pH, electrical conductivity, and water holding capacity were described by Mir et al. [21], who emphasized that these traits are strongly associated with the technological quality of poultry meat. The effect of sex on the analyzed traits was limited; however, the lower pH and lower cooking loss of breast muscles in female Muscovy ducks may indicate better water-holding capacity of the meat from females.

The present study demonstrated that duck genotype significantly affected the color of breast and leg muscles, which was reflected by higher L*, a*, and b* values in Pekin ducks compared with Muscovy ducks. The lighter color of meat (higher L*) and higher redness and yellowness values in Pekin ducks may be associated with a greater intramuscular fat content and a different muscle fiber structure. Similar relationships were described by Mir et al. [21], who indicated that fat content, muscle acidification, and postmortem changes significantly determine poultry meat color. Lower L* and b* values observed in Muscovy ducks suggest darker meat, which may result from higher metabolic activity of muscles and a greater proportion of red muscle fibers, more typical for this genotype. According to Kokoszyński et al. [5], the darker coloration of Muscovy duck breast muscles, compared with those of Pekin ducks, may be related to a better blood supply to the muscles in older Muscovy ducks. In contrast, the effect of sex was limited and concerned only selected color parameters, which may indicate that these traits are more strongly determined by genotype than by sex, which is consistent with the results of our previous studies [19,45].

Analysis of breast muscle texture traits revealed higher Warner–Bratzler shear force, hardness, and gumminess values in Pekin ducks, indicating a more compact muscle structure compared with Muscovy ducks. In contrast, higher springiness values observed in Muscovy ducks may indicate a more elastic muscle tissue structure, which is often associated with differences in muscle fiber composition and lower fatness. Similar relationships between genotype and poultry meat texture were described by Baéza et al. [27] and Mir et al. [21], who indicated that muscle fiber structure, growth rate, and postmortem changes significantly affect meat hardness and mechanical properties. The lack of a sex effect on texture traits may result from the small differences in the chemical composition of breast muscles between males and females at the same slaughter age, which limited the differentiation of meat mechanical properties. Similar observations were presented by Le Bihan-Duval et al. [22], who indicated that poultry meat texture is mainly determined by genotype and growth rate rather than by sex.

The obtained results indicate that duck genotype significantly affected the weight and percentage share of internal organs, which is consistent with observations regarding anatomical and physiological differences between Pekin and Muscovy ducks. The higher liver and gizzard weights observed in Pekin ducks may be associated with their more intensive growth rate and higher metabolic demands related to the rapid deposition of muscle and fat tissue. Similar relationships between genotype and internal organ weight in ducks were described by Andrieux et al. [47], who indicated that differences in growth rate and metabolism are reflected in the development of visceral organs. In Muscovy ducks, the higher relative proportion of some organs (e.g., the heart, spleen, and proventriculus) is consistent with the observations of Makram et al. [41], who demonstrated significant differences in the development of internal organs between Pekin and Muscovy ducks resulting from different growth dynamics and genetic background. The effect of sex on the analyzed traits may result from hormonal differences and different growth rates of males and females. Similar relationships were described by Houessionon et al. [43], who indicated a significant effect of sexual dimorphism on the development of internal organs in ducks.

Morphometric analysis of the digestive tract demonstrated greater lengths and diameters of most intestinal segments in Pekin ducks. Similar relationships were observed by Wasilewski et al. [31] and Chen et al. [30], who showed that faster-growing bird genotypes are characterized by a more developed digestive tract, enabling more efficient nutrient intake and utilization. The larger intestinal dimensions in Pekin ducks may be associated with their more intensive growth rate and higher feed intake. A larger digestive tract may increase the absorptive surface area and improve nutrient utilization efficiency, supporting the rapid growth characteristic of Pekin ducks. The development of the gastrointestinal tract is closely associated with nutrient demand, and adaptive enlargement of intestinal segments may represent a physiological response to higher metabolic requirements. The effect of sex on the length of individual intestinal segments indicates the occurrence of sexual dimorphism in digestive system development. Longer intestinal segments in males may result from their greater body weight and higher metabolic requirements. Similar observations were reported by Wegner et al. [19], who demonstrated greater digestive tract development in male ducks compared with females.

A limitation of the present study is that ducks of different genotypes were evaluated at their respective commercial slaughter ages. Consequently, some differences observed in organ development, intestinal morphometry, and skeletal traits may reflect both genotype-specific characteristics and age-related physiological development. Therefore, the results should be interpreted with caution and primarily as a comparison of the two genotypes under practical production conditions.

The present study demonstrated both genotype and sex effects on skeletal system development in ducks, particularly in Muscovy ducks. Greater values of selected tibia (GL, AL, and DD) and femur (GL, ML, GD, and GE) parameters in female Pekin ducks may be associated with their faster growth rate and more intensive skeletal development. Similar relationships were observed by Włodarczyk et al. [48] and Wegner et al. [19], who demonstrated a significant effect of genotype on the dimensions and development of limb bones in ducks. The effect of sex was particularly evident in Muscovy ducks, where males were characterized by larger dimensions of most analyzed tibia and femur traits. This may result from the pronounced sexual dimorphism typical of this genotype and the greater body weight of males. Similar observations were presented by Wilkiewicz-Wawro et al. [44], who indicated more intensive skeletal system development in male Muscovy ducks.

## 5. Conclusions

This study demonstrated that both genotype and sex significantly influence carcass traits, meat quality, and the development of internal organs and the skeletal system in Muscovy and Pekin ducks. The results indicate clear differences in production efficiency and meat quality between the two genotypes, with Muscovy ducks showing traits associated with higher lean meat yield and lower fat deposition, while Pekin ducks were characterized by higher intramuscular fat content and different technological properties of their meat. These findings highlight the importance of considering both genotype and sex in duck production systems to optimize carcass value and meat quality. The results may be useful for breeding and management strategies aimed at improving production efficiency and product quality in commercial duck farming.

## Figures and Tables

**Table 1:** Carcass weight and proportion of carcass elements in the carcass weight of Muscovy and Pekin ducks at market age.

Trait	Muscovy		Pekin		SEM	*p*-Values		
	Male (n = 10)	Female (n = 10)	Male (n = 10)	Female (n = 10)		B	S	B × S
Body weight (g)	4000.0	2437.5 *	3298.8	3096.3	99.92	0.434	0.003	0.003
Carcass weight (g)	2823.2	1693.8 *	2276.5	2131.7 *	65.8	0.060	0.003	0.003
Carcass yield (%)	70.6	69.5	69.0	68.9	0.36	0.060	0.268	0.570
Wings with skin (%)	16.2 ^a^	17.4 ^a^	13.0 ^b^	14.0 ^b^	0.35	0.002	0.066	0.944
Neck without skin (%)	5.8 ^b^	5.0 ^b^*	7.8 ^a^	7.5 ^a^*	0.21	0.002	0.031	0.280
Pectoral muscle (%)	18.2	17.7	17.5	16.3	0.29	0.122	0.241	0.685
Leg muscles (%)	12.6	10.0	12.0	12.5	0.31	0.128	0.062	0.032
Skin with fat (%)	17.1	20.2	20.8	20.5	0.36	0.051	0.066	0.023
Abdominal fat (%)	0.8	0.9	1.0	1.0	0.06	0.434	0.850	0.867
Remainders (%)	29.3	28.8	27.9	28.2	0.56	0.083	0.552	0.583

^a,b^ are the means with different superscripts that are statistically different between breeds within a given gender (*p* < 0.05). * statistical differences between males and females (*p* < 0.05) within a given breed. B, breed; S, sex; B × S, interaction.

**Table 2:** Basic chemical composition of Muscovy and Pekin duck meat at market age.

Trait		Muscovy		Pekin		SEM	*p*-Values		
		Male (n = 10)	Female (n = 10)	Male (n = 10)	Female (n = 10)		B	S	B × S
Intramuscular fat (%)	PM	1.2 ^b^	1.7 ^b^	2.4 ^a^	2.1 ^a^	0.11	0.002	0.111	0.008
	LM	3.4 ^b^	4.4 ^b^*	5.7 ^a^	6.1 ^a^	0.24	0.002	0.003	0.003
Protein (%)	PM	22.9	22.5	23.1	23.5	0.12	0.122	0.110	0.003
	LM	21.7 ^a^	21.5	21.0 ^b^	21.5	0.30	0.013	0.529	0.225
Collagen (%)	PM	1.3	1.2 ^b^	1.4	1.4 ^a^	0.02	0.004	0.161	0.289
	LM	1.3 ^b^	1.7	1.8 ^a^	1.6	0.04	0.018	0.803	0.003
Water (%)	PM	72.1	72.0	71.1	71.8	0.16	0.360	0.447	0.468
	LM	71.1 ^a^	71.5 ^a^	68.6 ^b^	69.8 ^b^*	0.33	0.002	0.003	0.003

^a,b^ are the means with different superscripts that are statistically different between breeds within a given gender (*p* < 0.05). * statistical differences between males and females (*p* < 0.05) within a given breed. PM, pectoral muscle; LM, leg muscle; B, breed; S, sex; B × S, interaction.

**Table 3:** Some physicochemical parameters of Muscovy and Pekin duck meat at market age.

Trait		Muscovy		Pekin		SEM	*p*-Values		
		Male (n = 10)	Female (n = 10)	Male (n = 10)	Female (n = 10)		B	S	B × S
pH_24 hs_	PM	5.98	5.70 *	5.89	5.92	0.02	0.638	0.077	0.023
	LM	6.21	6.09 ^b^	6.22	6.27 ^a^	0.02	0.049	0.529	0.131
EC_24 hs_—(mS/cm)	PM	10.2	10.8	9.0	9.1	0.24	0.192	0.416	0.379
	LM	11.5 ^a^	9.9 ^a^	9.0 ^b^	9.1 ^b^	0.22	0.018	0.062	0.042
Thermal loss (%)	PM	31.2 ^b^	28.7 ^b^*	36.9 ^a^	36.1 ^a^	0.62	0.002	0.035	0.288
	LM	30.6 ^b^	29.8 ^b^	38.5 ^a^	36.1 ^a^	0.84	0.002	0.417	0.677

^a,b^ are the means with different superscripts that are statistically different between breeds within a given gender (*p* < 0.05). * statistical differences between males and females (*p* < 0.05) within a given breed. PM, pectoral muscle; LM, leg muscle; B, breed; S, sex; B × S, interaction; EC, electrical conductivity.

**Table 4:** Color parameters of Muscovy and Pekin duck meat at market age.

Trait		Muscovy		Pekin		SEM	*p* Values		
		Male (n = 10)	Female (n = 10)	Male (n = 10)	Female (n = 10)		B	S	B × S
L*—lightness	PM	39.4 ^b^	33.9 ^b^*	44.3 ^a^	44.4 ^a^	0.77	0.002	0.006	0.006
	LM	40.2 ^b^	41.6 ^b^	46.0 ^a^	43.9 ^a^	0.68	0.010	0.837	0.303
a*—redness	PM	16.2 ^b^	17.7 ^b^*	19.4 ^a^	19.7 ^a^	0.26	0.002	0.028	0.144
	LM	13.4 ^b^	13.7 ^b^	19.6 ^a^	17.7 ^a^	0.54	0.002	0.440	0.280
b*—yellowness	PM	2.7 ^b^	3.4 ^b^	7.1 ^a^	5.8 ^a^	0.37	0.002	0.701	0.151
	LM	3.6 ^b^	3.0 ^b^*	6.9 ^a^	4.4 ^a^*	0.37	0.019	0.054	0.265

^a,b^ are the means with different superscripts that are statistically different between breeds within a given gender (*p* < 0.05). * statistical differences between males and females (*p* < 0.05) within a given breed. PM, pectoral muscle; LM, leg muscle; B, breed; S, sex; B × S, interaction.

**Table 5:** Textural features of the pectoralis major muscle of Muscovy and Pekin ducks of market age.

Trait	Muscovy		Pekin		SEM	*p*-Values		
	Male (n = 10)	Female (n = 10)	Male (n = 10)	Female (n = 10)		B	S	B × S
WB shear force (N)	50.7 ^b^	49.8 ^b^	66.4 ^a^	64.5 ^a^	1.74	0.002	0.731	0.921
Hardness (N)	32.7 ^b^	33.4 ^b^	45.8 ^a^	43.8 ^a^	1.24	0.002	0.803	0.969
Gumminess (N)	13.9 ^b^	13.6 ^b^	17.0 ^a^	16.1 ^a^	0.40	0.020	0.529	0.822
Cohesiveness	0.4	0.4	0.4	0.4	0.01	0.905	0.254	0.551
Springiness (cm)	1.5 ^a^	1.5 ^a^	1.1 ^b^	1.2 ^b^	0.03	0.002	0.529	0.606
Chewiness (N × cm)	20.1	19.9	19.1	19.0	0.46	0.418	0.875	0.999

^a,b^ are the means with different superscripts that are statistically different between breeds within a given gender (*p* < 0.05). B, breed; S, sex; B × S, interaction.

**Table 6:** Content of selected internal organs relative to pre-slaughter body weight of Muscovy and Pekin ducks at market age.

Trait		Muscovy		Pekin		SEM	*p*-Value		
		Male (n = 10)	Female (n = 10)	Male (n = 10)	Female (n = 10)		B	S	B × S
Gizzard	(g)	73.4 ^b^	73.1 ^b^	100.8 ^a^	99.4 ^a^	2.81	0.002	0.873	0.867
	(%)	1.8 ^b^	3.0 *	3.1 ^a^	3.2	0.12	0.002	0.003	0.003
Proventriculus	(g)	7.0	6.7	9.0	7.7 *	0.42	0.428	0.044	0.003
	(%)	0.18	0.27 *	0.27	0.25	0.02	0.071	0.003	0.003
Liver	(g)	53.8 ^b^	42.6 ^b^*	60.1 ^a^	58.0 ^a^	1.75	0.002	0.062	0.062
	(%)	1.3 ^b^	1.7 *	1.8 ^a^	1.9	0.05	0.002	0.009	0.150
Spleen	(g)	1.8 ^a^	1.2 *	1.2 ^b^	1.2	0.07	0.009	0.021	0.003
	(%)	0.05	0.05	0.04	0.04	0.01	0.096	0.837	0.516
Heart	(g)	21.4 ^a^	12.5 *	14.1 ^b^	13.5	0.69	0.002	0.003	0.003
	(%)	0.5 ^a^	0.5 ^a^	0.4 ^b^	0.4 ^b^	0.01	0.002	0.758	0.374

^a,b^ are the means with different superscripts that are statistically different between breeds within a given gender (*p* < 0.05). * statistical differences between males and females (*p* < 0.05) within a given breed. B, breed; S, sex. B × S, interaction.

**Table 7:** Intestine traits of Muscovy and Pekin ducks in the market age.

Trait	Muscovy		Pekin		SEM	*p*-Value		
	Male (n = 10)	Female (n = 10)	Male (n = 10)	Female (n = 10)		B	S	B × S
Lenght (cm)								
Duodenum	30.5 ^b^	24.8 ^b^*	39.2 ^a^	37.8 ^a^	1.26	0.002	0.047	0.248
Jejunum	84.9 ^b^	67.5 ^b^*	89.3 ^a^	84.6 ^a^*	1.78	0.002	0.003	0.011
Ileum	82.1 ^b^	64.9 ^b^*	88.9 ^a^	80.5 ^a^*	1.88	0.002	0.003	0.094
Caeca	37.2	26.4 ^b^*	39.4	36.1 ^a^	1.00	0.002	0.003	0.003
Colon	16.6 ^a^	11.6 *	13.5 ^b^	11.2 *	0.44	0.002	0.003	0.029
Total intestine	251.3 ^b^	195.2 ^b^*	270.3 ^a^	250.2 ^a^*	5.52	0.002	0.003	0.003
Diameter (mm)								
Duodenum	7.5 ^b^	8.8 ^b^*	9.8 ^a^	10.1 ^a^	0.25	0.002	0.043	0.360
Jejunum	7.0 ^b^	7.1 ^b^	8.8 ^a^	8.3 ^a^	0.21	0.002	0.529	0.526
Ileum	7.3 ^b^	7.9 ^b^	10.3 ^a^	9.6 ^a^	0.27	0.002	0.096	0.999
Caeca	7.0	6.6	7.5	7.4	0.17	0.106	0.529	0.867
Colon	8.9 ^b^	10.3 ^b^	15.3 ^a^	13.6 ^a^	0.49	0.002	0.829	0.003

^a,b^ are the means with different superscripts that are statistically different between breeds within a given gender (*p* < 0.05). * statistical differences between males and females (*p* < 0.05) within a given breed. B, breed; S, sex; B × S, interaction.

**Table 8:** Diameters of the femur and tibia bones of Muscovy and Pekin ducks in the market age.

Trait	Muscovy		Pekin		SEM	*p*-Value		
	Male (n = 10)	Female (n = 10)	Male (n = 10)	Female (n = 10)		B	S	B × S
Femur bone (mm)								
GL	84.5 ^a^	66.9 ^b^*	72.4 ^b^	70.8 ^a^	1.24	0.002	0.003	0.003
ML	79.2 ^a^	62.0 ^b^*	67.6 ^b^	66.8 ^a^	1.16	0.002	0.003	0.003
GB	23.0 ^a^	17.6 *	18.7 ^b^	18.3	0.41	0.002	0.003	0.003
GD	10.6 ^b^	8.4 ^b^*	12.7 ^a^	12.0 ^a^	0.33	0.002	0.021	0.042
SM	8.0	6.4 *	7.6	6.8 *	0.16	0.905	0.003	0.113
GC	20.7	16.0 *	19.3	18.9	0.36	0.120	0.003	0.003
GE	13.2 ^b^	11.3 ^b^*	18.6 ^a^	18.1 ^a^	0.56	0.002	0.003	0.003
Tibia bone (mm)								
GL	137.7 ^a^	111.0 ^b^*	120.7 ^b^	118.9 ^a^	1.81	0.002	0.003	0.003
AL	129.0 ^a^	104.2 ^b^*	112.8 ^b^	110.5 ^a^	1.69	0.002	0.003	0.003
GD	25.9	20.6 *	24.6	23.2 *	0.40	0.140	0.003	0.003
SB	6.2	6.0	5.7	5.6	0.12	0.106	0.731	0.865
SD	17.6	14.3 *	17.0	16.4	0.27	0.071	0.020	0.003
DD	14.5 ^b^	11.5 ^b^*	17.5 ^a^	16.9 ^a^	0.46	0.002	0.006	0.003

^a,b^ are the means with different superscripts that are statistically different between breeds within a given gender (*p* < 0.05). * statistical differences between males and females (*p* < 0.05) within a given breed. Femur bone dimensions: GL, greatest length; ML, medial length; GB, greatest breadth of proximal end; GD, greatest depth of proximal end; SM, smallest breadth of the corpus; GC, greatest breadth of the distal end; GE, greatest depth of distal end. Tibia bone dimensions: GL, greatest length; AL, axial length; GD, greatest diagonal of the proximal end; SB, smallest breadth of the corpus; SD, greatest breadth of the distal end; DD, greatest depth of the distal end; B, breed; S, sex; B × S, interaction.

## Data Availability

Data will be provided upon request.
